# Identification of autophagy-related genes in neuropathic pain through bioinformatic analysis

**DOI:** 10.1186/s41065-023-00269-w

**Published:** 2023-03-01

**Authors:** Sheng Tian, Lanxiang Wu, Heqing Zheng, Xianhui Zhong, Xinping Yu, Wei Wu

**Affiliations:** Department of Neurology, The Second Affiliated Hospital of Nanchang University, Nanchang, 330006 China

**Keywords:** Neuropathic pain, Autophagy, Bioinformatic analysis, Sirt2

## Abstract

**Background:**

Neuropathic pain (NP) is one of the most common types of chronic pain and significantly compromises the quality of life. Autophagy is an intracellular catabolic process that is required to maintain cellular homeostasis in response to various stresses. The role of autophagy-related genes in the diagnosis and treatment of neuropathic pain remains unclear.

**Methods:**

We identified autophagy-related differentially expressed genes (ARDEGs) and differentially expressed miRNAs (DE-miRNAs) in neuropathic pain by bioinformatics analysis of the GSE145226 and GSE145199 datasets. These ARDEGs and their co-expressed genes were subjected to Gene Ontology (GO), Kyoto Encyclopedia of Genes and Genomes (KEGG) enrichment analysis, Gene Set Enrichment Analysis (GSEA) and friends analysis. Meanwhile, we constructed TFs-ARDEGs, miRNA-ARDEGs regulatory network through ChIPBase database and the HTFtarget database, multiMir R package. Finally, we performed immune infiltration analysis of ARDEGs by Single Sample Gene Set Enrichment Analysis (ssGSEA).

**Results:**

We identified 2 potential autophagy-related differentially expressed genes (Sirt2 and ST7) that may be closely associated with the pathogenesis of neuropathic pain. GO, KEGG and GSEA analysis revealed that these two ARDEGs were mainly enriched in pyridine nucleotide metabolic process, nicotinamide nucleotide metabolic process, Nicotinate and nicotinamide metabolism, NF-κB pathway, KRAS signaling, P53 pathway. In the TFs-ARDEGs and miRNA-ARDEGs regulatory network, miR-140-5p and Cebpb were predicted to be as crucial regulators in the progression of NP. For the ssGSEA results, Sirt2 was positively correlated with Eosinophil and Effector memory CD8^+^ T cell infiltration, which suggested that it may be involved in the regulation of neuroimmune-related signaling.

**Conclusion:**

Two autophagy-related differentially expressed genes, especially Sirt2, may be potential biomarkers for NP, providing more evidence about the crucial role of autophagy in neuropathic pain.

**Supplementary Information:**

The online version contains supplementary material available at 10.1186/s41065-023-00269-w.

## Background

Neuropathic pain (NP) is a common pain syndrome with primary clinical manifestations, including allodynia, spontaneous pain and hyperalgesia [1]. And patients with neuropathic pain often have sleep disorders, depression, and anxiety. According to previous study, at least 1%-5% of the individuals worldwide were diagnosed with neuropathic pain each year [2]. At present, the incidence of NP continues to increase, and the medical expenses increase year by year, which has become one of the major public health problems in the world [3]. The course of NP is prolonged and requires long-term treatment, but so far there is still a lack of effective treatment. Conventional nerve blocks, surgical operations, and analgesic drugs are not always effective, and about half of the patients cannot adequately relieve pain, accompanied by different degrees of adverse reactions [4]. Considering the intractable and economic burden of NP, disentangling the pathogenesis, and finding effective treatment are urgently needed.

Autophagy, which is a physiological process that degrades self-damaged organelles and macromolecules through lysosomes, is critical for maintaining homeostasis in the intracellular environment, survival, differentiation, development [5]. Autophagy is regulated by a group of autophagy related genes (ARGs) and recognized by corresponding proteins. These proteins form autophagosomes with a bilayer membrane structure and then fuse with lysosomes to perform relevant functions [6]. Some studies have shown that autophagy is involved in pathological processes associated with central nervous system diseases such as Alzheimer's disease, cerebral ischemia, and spinal cord injury [7, 8]. In addition, autophagy is involved in immune defense and inflammatory regulatory processes, which have received extensive attention [9]. For example, autophagy can regulate inflammasome-dependent responses by controlling the level of pro-inflammatory cytokine secretion [10]. When autophagy was activated by inflammatory signals, cytokine production became limited. A study has shown that inflammatory and immune mechanisms in the peripheral and central nervous systems contribute to the development of neuropathic pain [11]. Infiltration of inflammatory cells and activation of innate immune cells after nerve injury result in the production and secretion of inflammatory mediators. These inflammatory mediators rapidly activate neuroimmunity, sensitize primary afferent neurons, and cause hyperalgesia [12]. Thus, autophagy may be involved in the progression of neuropathic pain through the modulation of immune responses. Autophagy can also be used as a tool to assess the effectiveness of experimental therapeutic interventions targeting neuropathic pain. Shi G et al. have found that the miRNA-195 increased neuroinflammation and neuropathic pain by inhibiting autophagy activation following peripheral nerve injury [13]. The study also found that miR-195 inhibitor treatment increased autophagy activation and suppressed neuroinflammation. However, the molecular mechanisms underlying the interaction of neuropathic pain and autophagy is unclear.

With the development of molecular biology and next-generation sequencing technologies, it has become possible to explore the underlying mechanisms of disease at the genetic and RNA levels on a large scale [14]. By comparing disease cohorts with normal control cohorts, we can obtain a large number of gene expression profiles and identify differentially expressed genes associated with the development and progression of disease. For instance, Simin Tang et al. identified key transcription factors (MEF2A) and microRNAs (miR‐16‐5p) in the dorsal root ganglion through bioinformatic analysis, and these two molecules were crucial regulators in the progression of NP [15]. However, bioinformatics analyses exploring the relationship between neuropathic pain and autophagy are scarce.

Recently, researchers have suggested that neuropathic pain behaviors are associated with synaptic plasticity and limbic cortical alteration [16]. However, in contrast to the limbic system, previous studies on key markers of neuropathic pain mostly focused on inflammatory changes in spinal cord neurons. Therefore, we selected two gene datasets of limbic cortex for analysis to identify key biomarkers of neuropathic pain. Furthermore, this study explored the relationship between autophagy-related genes and NP at the gene level, constructed related regulatory networks, revealed potential therapeutic agents and obtained information on the correlation between key autophagy-related genes and immune infiltrating cells. The results of this study may provide a reference for autophagy as a therapeutic target for NP and these autophagy-related biomarkers may be used for disease diagnosis and treatment monitoring.

## Materials and methods

### Data collection

Neuropathic pain-related microarray data (GSE145199 and GSE145226) were collected from the Gene Expression Omnibus (GEO) database (http://www.ncbi.nlm.nih.gov/geo/) via the GEOquery R package [17, 18]. The miRNA expression profiles were obtained from GSE145199 (3 pairs of rat nerve injury samples and rat control samples) based on the platform of the GPL21572 [19]. The mRNA expression profiles were obtained from GSE145226 (3 pairs of SNI group samples and control samples) based on the platform of the GPL1355(19). In the GeneCards database (http://www.genecards.org/), 7822 autophagy-related genes (ARGs) were retrieved by the word "autophagy" (Table S1) [20].

### Analysis of differentially expressed genes

The neuropathic pain-related GSE145199 and GSE145226 datasets were first normalized using the limma R package [21]. We then used the limma R package to perform differential analysis of genes in different groups of GSE145226 and GSE145199 datasets. The genes screened by the criteria of |logFC|> 0 and *p* value < 0.05 were regarded as differentially expressed genes (DEGs) [22]. Differentially expressed miRNAs (DE-miRNAs) in the GSE145199 dataset were obtained by the same threshold. To obtain autophagy-related differentially expressed genes (ARDEGs) related to neuropathic pain, we intersected autophagy-related genes and differentially expressed genes (DEGs), and draw a Venn diagram. The results of differentially expressed genes were displayed by ggplot2 R package to draw volcano map and pheatmap R package to draw heat map.

### PPI network construction

Protein–protein interaction (PPI) network is composed of proteins and proteins through their interactions with each other, which participate in biological signal transmission, gene expression regulation, energy, material metabolism, and cell cycle regulation [23]. The STRING database is a database for searching interactions between known and predicted proteins [24]. In this study, we used the STRING database to take the minimum required interaction score greater than 0.900 as the criteria for ensuring accuracy, and took the top10 genes with the minimum required interaction score as the co-expression of ARDEGs [25, 26]. The ten co-expressed genes and autophagy-related differentially expressed genes were used to construct a PPI network, which was visualized using Cytoscape software [27].

### GO and KEGG enrichment analysis

Gene Ontology (GO) analysis is a common method for large-scale functional enrichment analysis, including biological process (BP), molecular function (MF) and cell component (CC) [28]. The Kyoto Encyclopedia of Genes and Genomes (KEGG) is a widely used database for storing information about genomes, pathways, diseases, and drugs [29].We used the clusterProfiler R package to perform GO annotation analysis and KEGG pathway enrichment analysis on ARDEGs and 10 co-expressed differentially expressed genes screened through the STRING database [30]. Item screening criteria were *p *value < 0.05 was considered statistically significant, and the *p* value correction method was Benjamini Hochberg (BH).

### Gene set enrichment analysis (GSEA)

Gene Set Enrichment Analysis (GSEA) is a computational method to analyze whether a specific gene set is statistically different between two biological states, and is usually used to estimate the activity of pathways and biological processes in expression dataset [31]. We performed GSEA on the neuropathic pain-related GSE145226 dataset using the clusterProfiler R package. The parameters used in this GSEA enrichment analysis were as follows: the seed was 2020, the number of computations was 10,000, the number of genes contained in each gene set was at least 10, the number of genes contained at most was 500, and the *p*-value correction method was Benjamini-Hochberg (BH). We obtained the h.all.v7.2.symbols.gmt gene set from the Molecular Signatures Database (MSigDB), and the screening criteria for significant enrichment was *p *value < 0.05 [32].

### Construction of TFs-ARDEGs, miRNA- ARDEGs, drugs- ARDEGs regulatory network

We explored the regulatory effects of transcription factors (TFs) on autophagy-related differentially expressed genes through the transcription factors retrieved from the ChIPBase database and the HTFtarget database [33, 34].

In order to analyze the regulatory relationship between autophagy-related differentially expressed genes and miRNAs, miRNAs related to ARDEGs were obtained from the multiMir R package, and the final results were obtained by intersecting them with DE-miRNAs [35, 36].

We used the Comparative Toxicogenomics Database (CTD) to predict the direct and indirect drug targets of ARDEGs to explore the interaction between ARDEGs and drugs [37]. Finally, the Cytoscape software was used to visualize the above regulatory network.

### Friends analysis

Semantic comparison of Gene Ontology (GO) annotation provides a quantitative method for similarity analysis between genes and genomes, and has become an important basis for many bioinformatics analysis methods. To further compare the similarity between ARDEGs and genomes, we used the GOSemSim R package to calculate ARDEGs in biological processes, molecular function and cell component levels of geometric mean value, and calculate the GO semantic similarity of ARDEGs to get the final score [38]. Finally, the results of the friends analysis were visualized using the ggplot R package.

### Single sample gene set enrichment analysis

Single sample gene set enrichment analysis (ssGSEA), an extension of the GSEA method, allows the definition of an enrichment score that represents the absolute degree of enrichment of the gene set in each sample within a specified dataset [39]. We used the ssGSEA algorithm to quantify the relative abundance of the GSE145226 dataset with twenty-eight immune cell infiltrations (Table S2). Meanwhile, we performed Spearman correlation analysis on the infiltration degree of various types of immune cells and ARDEGs in each sample of the GSE145226 dataset. The correlation analysis results were then visualized by using the pheatmap R package, and correlation scatter plots were drawn for statistically significant correlation results.

### Statistical analysis

All analyses in this study were based on R software (version 4.2.1). Continuous variables were presented as mean ± standard deviation. Comparisons between the two groups were performed using the Wilcoxon rank sum test. If there is no special indication, the results were calculated by the correlation coefficient between different molecules by Pearson correlation analysis. And all the results were taken the *P* value less than 0.05 as a statistically significant difference.

## Results

### Identification of DEGs, DE-miRNAs and autophagy-related DEGs

**Fig. 1:**
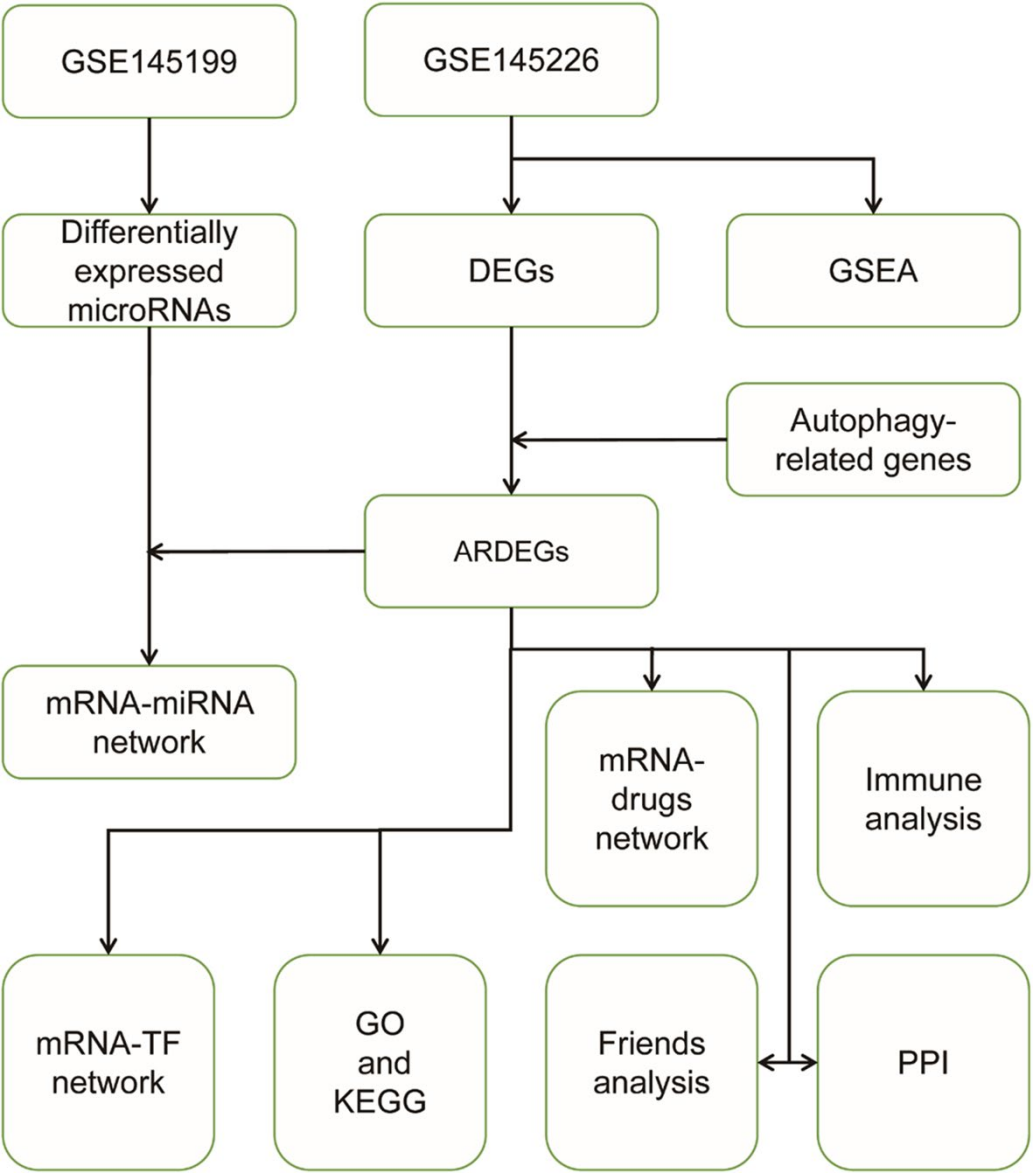
Study flowchart

**Fig. 2:**
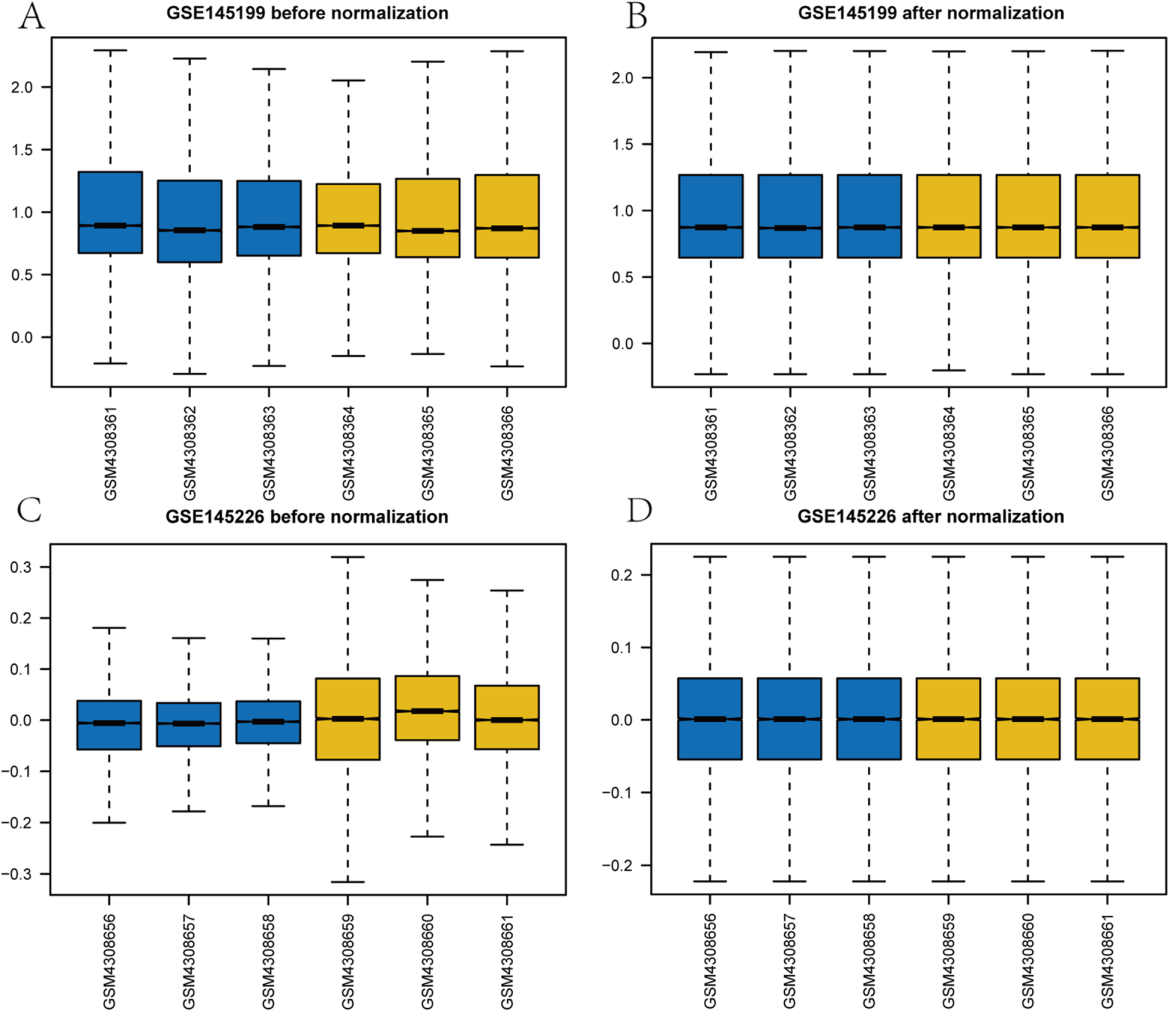
Data normalization. **A** GSE145199 dataset before normalization. **B** GSE145199 dataset after normalization. **C** GSE145226 dataset before normalization. **D** GSE145226 dataset after normalized. The blue color represents the sample of the SNI group. Yellow represents the sample of control group

**Fig. 3:**
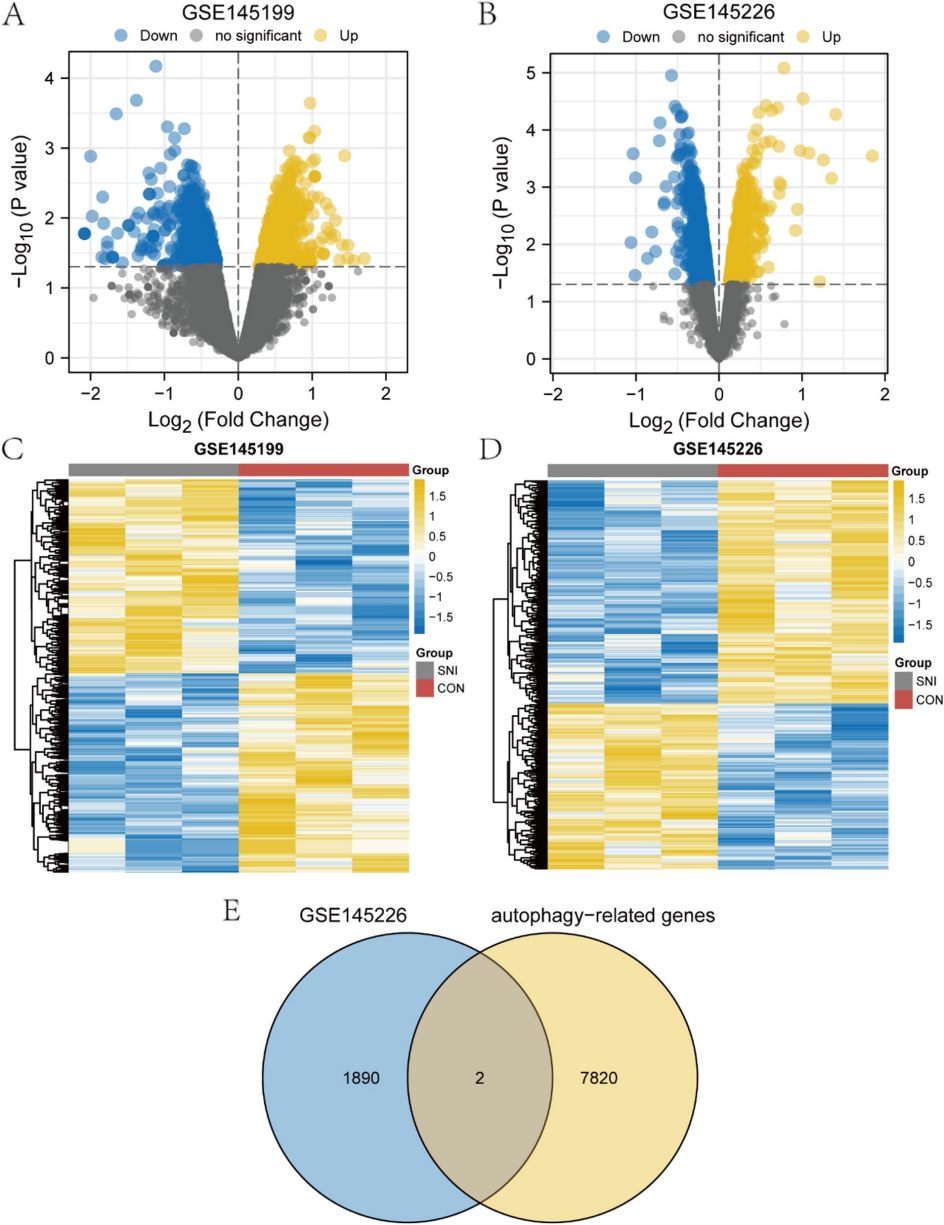
Identification of DEGs, DE-miRNAs and ARDEGs. **A**, **C** The DE-miRNAs between SNI group and control group in the GSE145119 dataset. **B**, **D** The DEGs between SNI group and control group in the GSE145226 dataset. **E** The Autophagy-related DEGs between SNI group and normal control group in the GSE145226 dataset

The flow chart of this study is depicted in Fig. 1. We firstly normalized the GSE145199 dataset and GSE145226 dataset and plotted boxplots to show gene expression profiles before and after processing (Fig. 2A-D). The black lines are almost on the same line, indicating excellent standardisation, which also ensures the accuracy of subsequent data analysis (Fig. 2B, D). Subsequently, differential analysis was performed on the GSE145199 and GSE145226 dataset using the limma R package to obtain the differentially expressed genes of the two sets. A total of 1701 DE-miRNAs were obtained from the GSE145199 dataset, including 839 DE-miRNAs with up-regulated expression and 862 DE-miRNAs with down-regulated expression, as shown by the volcano plot (Fig. 3A). And 1892 DEGs were found to reveal a significant differential expression in the GSE145226 dataset. Compared with the control group, 806 DEGs were up-regulated and 1086 DEGs were down-regulated in the nerve injury group, as shown by the volcano plot (Fig. 3B). In addition, the heatmap showed that the expression profiles of dysregulated genes in both datasets are divided into different units (Fig. 3C-D). We intersected the DEGs in the GSE145226 dataset with autophagy-related genes, and finally obtained two ARDEGs, as shown by the Venn diagram (Fig. 3E). These results suggested that differentially expressed autophagy-associated genes identified from the microarray dataset may be able to accurately distinguish between SNI and control samples.

### PPI network and TFs-ARDEGs, miRNA- ARDEGs, drugs-ARDEGs regulatory network analysis

**Fig. 4:**
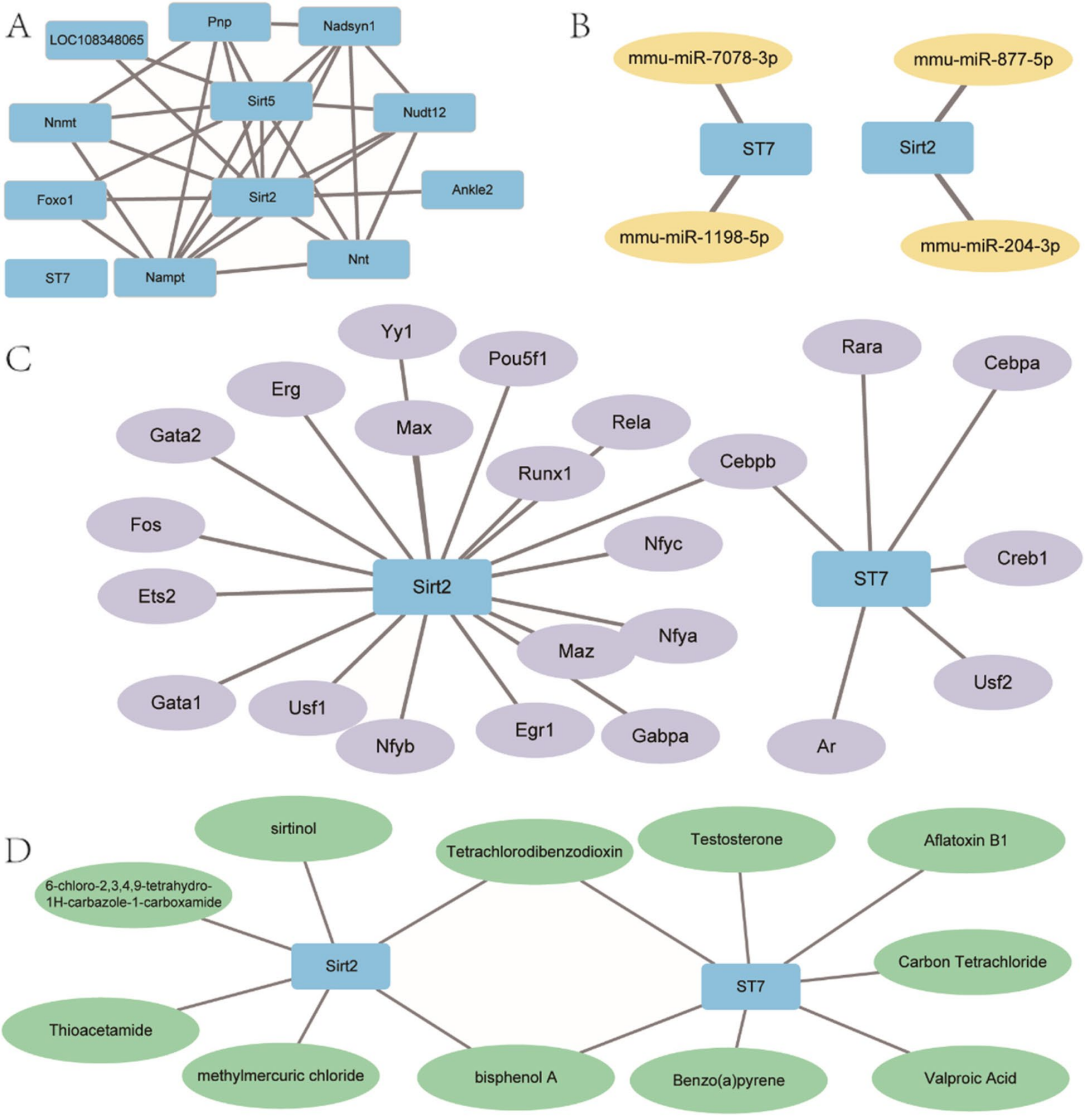
PPI Network, TFs-ARDEGs, miRNA- ARDEGs, drugs- ARDEGs interaction network. **A** The protein–protein interaction network of ARDEGs. **B** The miRNA-mRNA interaction network of ARDEGs. **C** The TFs-mRNA interaction network of ARDEGs. **D** The drugs- ARDEGs interaction network of ARDEGs

We performed protein–protein interaction analysis on autophagy-related differentially expressed genes, and used the STRING database to identify top10 genes as the co-expressed genes of ARDEGs. Then ARDEGs and 10 co-expressed genes (Nnt, Ankle2, Nudt12, Nadsyn1, Sirt5, Nampt, Pnp, LOC108348065, Foxo1, Nnmt) were used to construct a protein–protein interaction network (Fig. 4A).

The miRNAs related to ARDEGs were obtained from the multiMir R package and intersected with the differentially expressed miRNAs obtained from the GSE145199 dataset. A total of 2 miRNAs (rno-miR-140-5p, rno-miR-877) were obtained, and the miRNA-mRNA regulatory network was constructed (Fig. 4B).

Transcription factors (TFs) targeting autophagy-related differentially expressed genes were obtained through ChIPBase database and HTFtarget database. A total of 22 transcription factors were found, and an TFs- ARDEGs regulatory network was constructed (Fig. 4C).

Finally, we used the CTD database to identify potential drugs or molecular compounds (drugs) targeting ARDEGs. A total of 11 potential drugs or molecular compounds (bisphenol A, methylmercuric chloride, Thioacetamide, 6-chloro-2,3,4,9-tetrahydro-1H-carbazole-1-carboxamide, Sirtinol, Tetrachlorodibenzodioxin, Benzo(a)-pyrene, Aflatoxin B1, Valproic acid, Carbon Tetrachloride, Testosterone) were found. And the drugs-mRNA regulatory network was established (Fig. 4D). Therefore, Sirt2 may play a critical role in the development of neuropathic pain through PPI network analysis. Key miR-140-5p and Cebpb were predicted to be as crucial regulators in the progression of NP with miRNA‐mRNA regulatory network and TFs-mRNA regulatory network analysis. The most notable of these is Valproic acid, for which there is now scientific evidence to support its effectiveness as a therapeutic compound for the treatment of neuropathic pain [40].

### GO and KEGG enrichment analysis of ARDEGs

We performed GO and KEGG enrichment analysis on ARDEGs and 10 co-expressed genes.

**Fig. 5:**
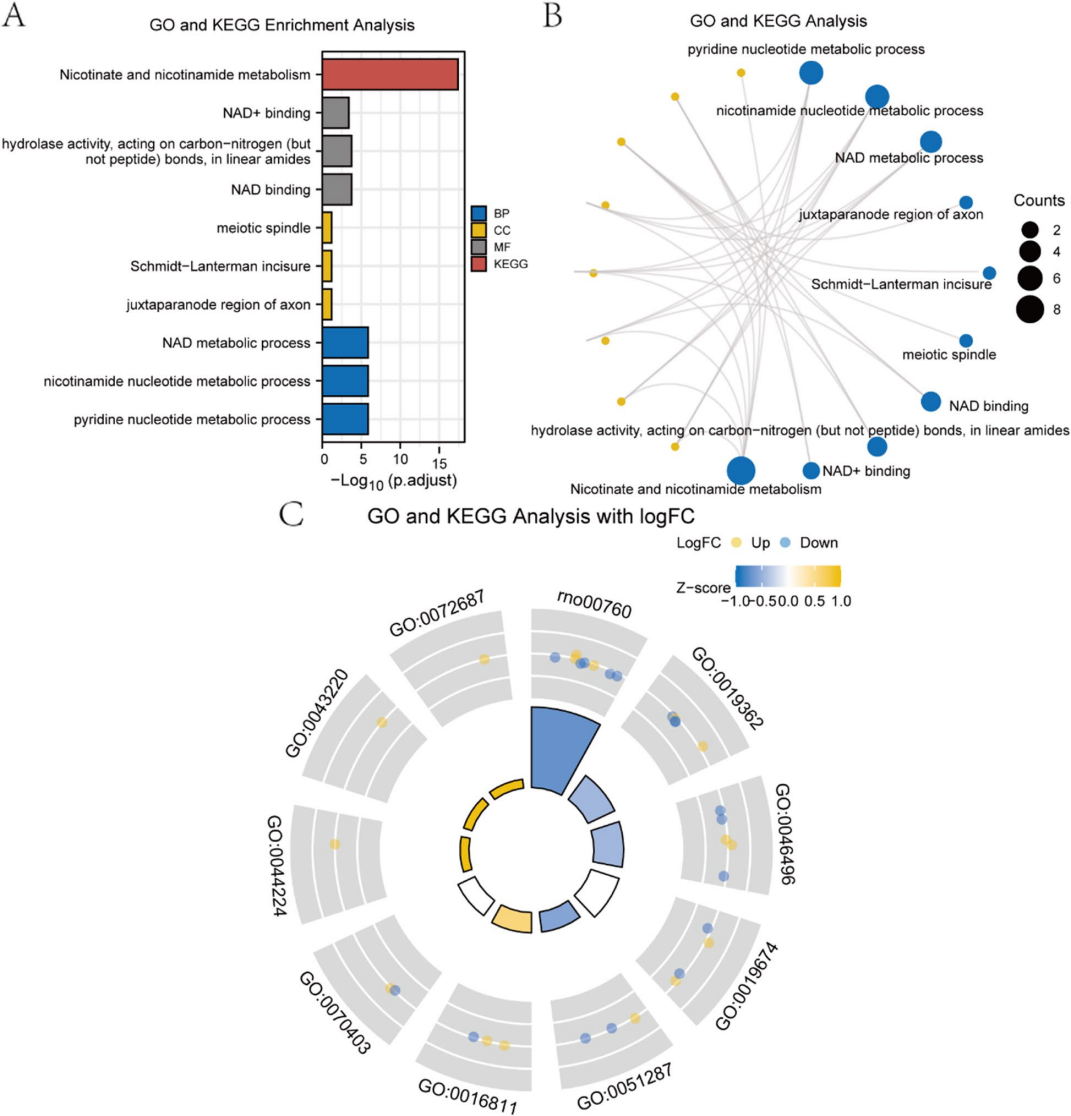
GO and KEGG enrichment analysis for ARDEGs. **A** Histogram of GO and KEGG enrichment analysis of ARDEGs. **B** Network diagram of GO and KEGG enrichment analysis of ARDEGs. **C** Circle diagram of combined logFC-based GO and KEGG enrichment analysis

As shown in Table 1, the enriched GO terms and enriched pathway were listed. According to the GO-BP category analysis, these genes were mainly related to pyridine nucleotide metabolic process, nicotinamide nucleotide metabolic process; cellular components such as juxtaparanode region of axon, Schmidt-Lanterman incisure. The GO-CC items showed that these genes were mainly located in juxtaparanode region of axon, Schmidt-Lanterman incisure. According to the GO-MF category analysis, these genes were involved in hydrolase activity, acting on carbon–nitrogen (but not peptide) bonds, in linear amides, NAD + binding. In our pathway enrichment analyses, the differential proteins were mainly related to Nicotinate and nicotinamide metabolism. In addition, we also visualized the results of GO functional enrichment analysis and KEGG pathway enrichment analysis through histograms (Fig. 5A). Meanwhile, a network diagram was drawn based on GO and KEGG enrichment analysis (Fig. 5B).

**Table 1:** GO terms and KEGG pathways for the ARDEGs and co-expressed genes

Ontology	ID	Description	*P* value
BP	GO:0019362	pyridine nucleotide metabolic process	9.26e-09
BP	GO:0046496	nicotinamide nucleotide metabolic process	9.26e-09
BP	GO:0019674	NAD metabolic process	9.31e-09
BP	GO:0072524	pyridine-containing compound metabolic process	1.13e-08
BP	GO:0006733	oxidoreduction coenzyme metabolic process	1.54e-08
CC	GO:0044224	juxtaparanode region of axon	0.005
CC	GO:0043220	Schmidt-Lanterman incisure	0.008
CC	GO:0072687	meiotic spindle	0.008
CC	GO:0033270	paranode region of axon	0.009
CC	GO:0043218	compact myelin	0.010
MF	GO:0051287	NAD binding	7.35e-06
MF	GO:0016811	hydrolase activity, acting on carbon–nitrogen (but not peptide) bonds, in linear amides	8.38e-06
MF	GO:0070403	NAD + binding	2.86e-05
MF	GO:0016810	hydrolase activity, acting on carbon–nitrogen (but not peptide) bonds	4.05e-05
MF	GO:0051721	protein phosphatase 2A binding	2.56e-04
KEGG	rno00760	Nicotinate and nicotinamide metabolism	2.23e-19

Finally, the combined logFC-based GO and KEGG enrichment analysis were performed on the expression profile data of the 12 genes in the dataset GSE145226. The standard score (Z-score) corresponding to each term was calculated using the logFC of the molecule and visualized by a circle diagram (Fig. 5C). Based on the GO and KEGG enrichment results, it suggested that ARDEGs may play an important role in the pathogenesis of neuropathic pain through these pathways and could be used for further analysis.

### Results of GSEA

**Fig. 6:**
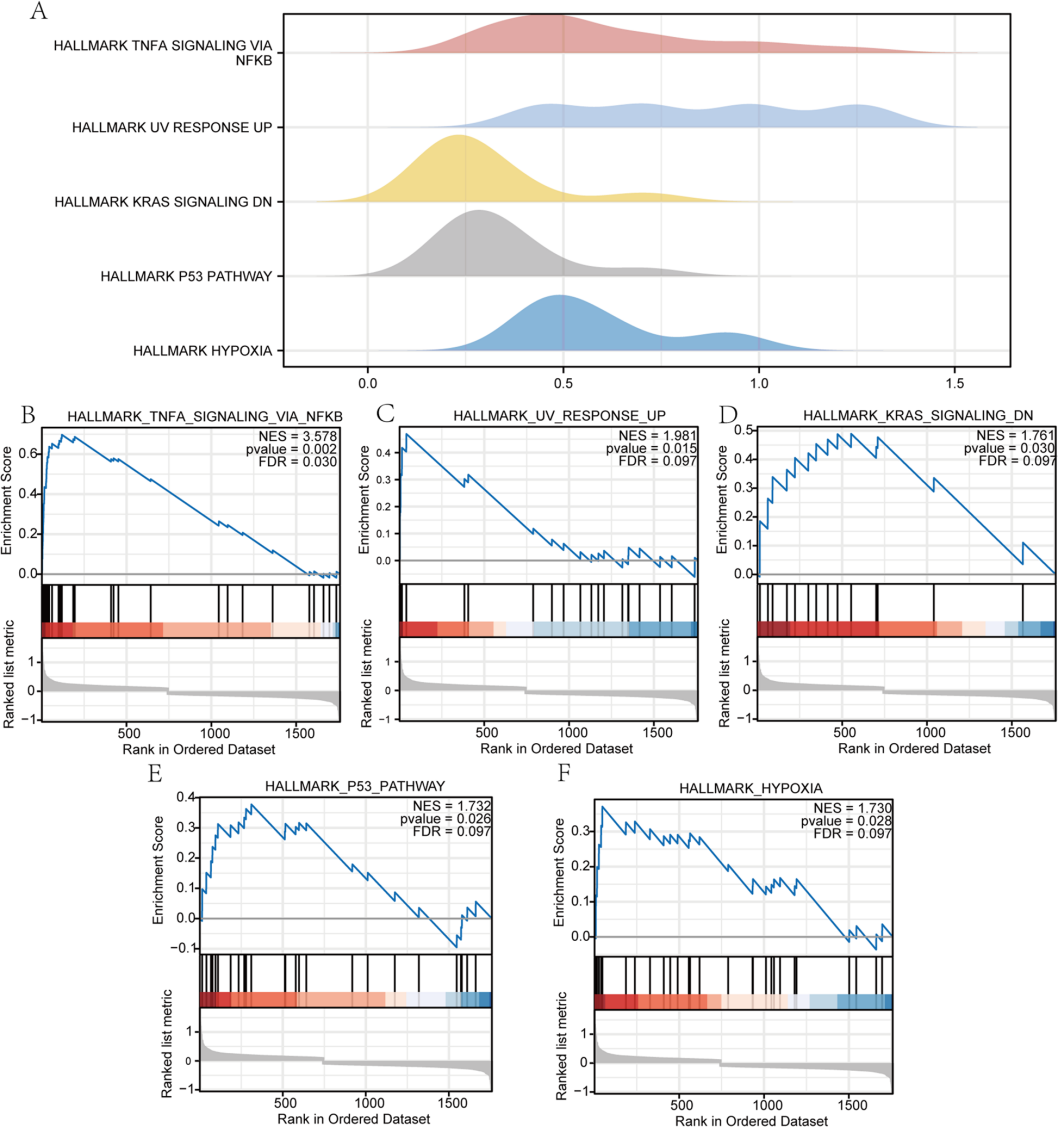
Results of GSEA enrichment analysis. **A** ridge plot of top5 pathways. **B**, **C**, **D**, **E**, **F** enrichment plot of top5 pathways

We further performed GSEA on the gene expression profiling in the GSE145226 dataset to discover signaling pathways that are differentially active in neuropathic pain. As shown in Table 2, a total of 20 crucial signaling pathways were obtained. The top5 pathways were drawn into a ridge plot (Fig. 6A). And the results showed that DEGs in the dataset GSE145226 were significantly enriched in NF-κB pathway (Fig. 6B), Uv response up (Fig. 6C), KRAS signaling (Fig. 6D), P53 pathway (Fig. 6E), Hypoxia (Fig. 6F). Thus, results of GSEA showed that NF-κB pathway, KRAS signaling, Hypoxia and P53 pathway were significantly more expressed in the SNI group and less expressed in the control group, suggesting that these pathways may be closely related to the pathogenesis of NP.

**Table 2:** GSEA for the genes

ID	Setsize	EnrichmentScore	NES	*P* value
HALLMARK_OXIDATIVE_PHOSPHORYLATION	42	-0.4283	-2.3908	0.002
HALLMARK_TNFA_SIGNALING_VIA_NFKB	36	0.6969	3.578	0.002
HALLMARK_UV_RESPONSE_UP	20	0.4691	1.9815	0.0146
HALLMARK_E2F_TARGETS	22	-0.3983	-1.7589	0.0156
HALLMARK_MYC_TARGETS_V1	39	-0.314	-1.6959	0.0204
HALLMARK_PI3K_AKT_MTOR_SIGNALING	16	-0.4478	-1.7707	0.0226
HALLMARK_P53_PATHWAY	25	0.3777	1.7325	0.0259
HALLMARK_HYPOXIA	27	0.371	1.7302	0.0281
HALLMARK_KRAS_SIGNALING_DN	14	0.4893	1.7608	0.0297
HALLMARK_KRAS_SIGNALING_UP	31	0.3158	1.5345	0.0644
HALLMARK_INFLAMMATORY_RESPONSE	19	0.3799	1.5533	0.0679
HALLMARK_FATTY_ACID_METABOLISM	22	-0.349	-1.541	0.0702
HALLMARK_ESTROGEN_RESPONSE_EARLY	22	0.3411	1.488	0.0798
HALLMARK_INTERFERON_GAMMA_RESPONSE	20	0.3521	1.4874	0.0917
HALLMARK_APICAL_JUNCTION	23	-0.3279	-1.4461	0.097
HALLMARK_PROTEIN_SECRETION	16	-0.36	-1.4237	0.1
HALLMARK_HEME_METABOLISM	17	0.3729	1.4474	0.1066
HALLMARK_DNA_REPAIR	21	-0.3269	-1.4168	0.1207
HALLMARK_UV_RESPONSE_DN	23	0.3126	1.3865	0.1247
HALLMARK_MYOGENESIS	29	0.2791	1.3241	0.1549

### Friends analysis of ARDEGs and their co-expressed genes

**Fig. 7:**
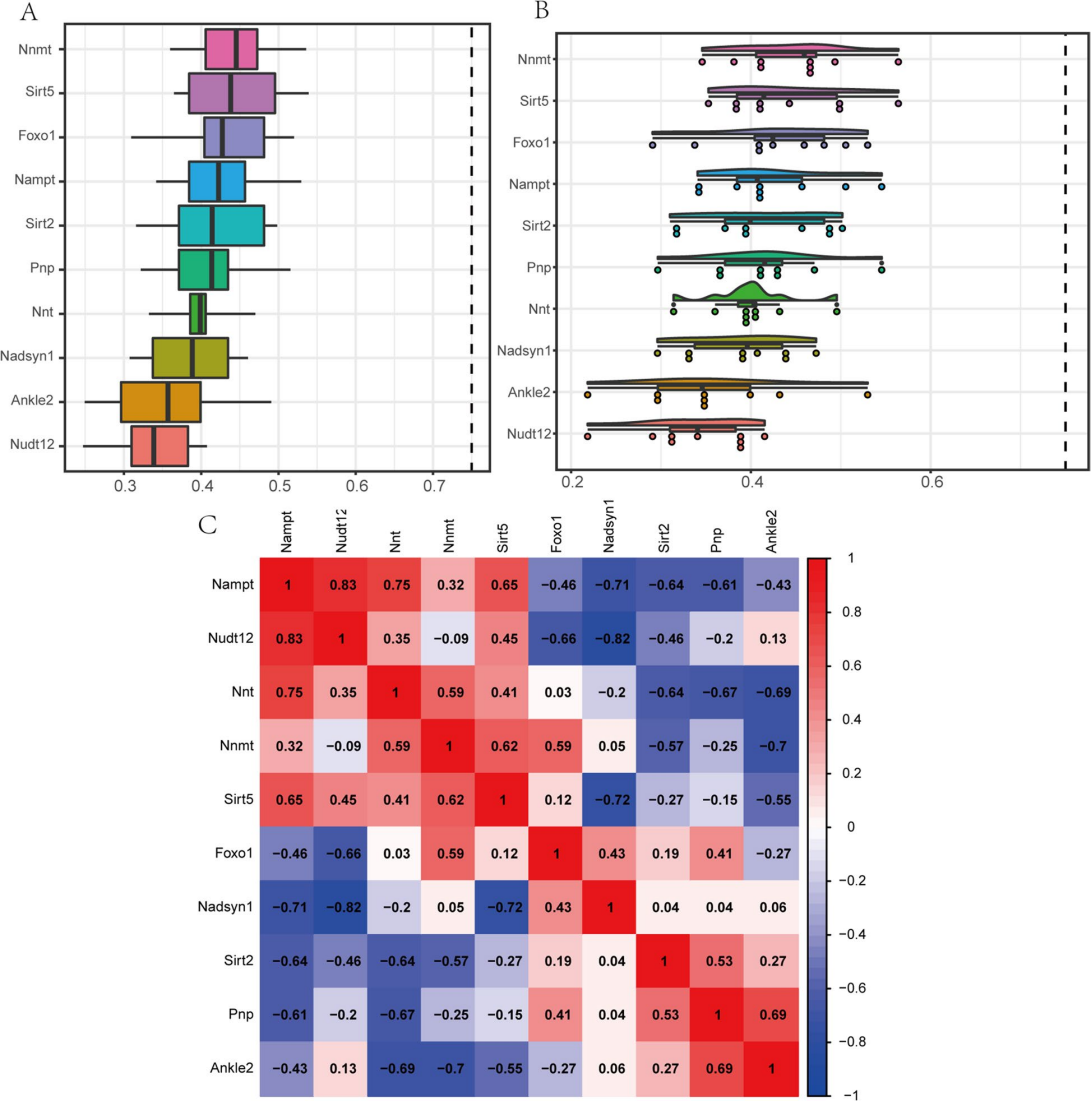
Friends analysis of ARDEGs. **A** Histogram of friends analysis of ARDEGs. **B** Cloud and rain map of friends analysis of ARDEGs. **C** Correlation heatmap of correlation analysis between gene expression level of ARDEGs in GSE145226 dataset

In order to compare the importance of ARDEGs in the pathway, we performed Friends analysis on Sirt2 and their co-expressed genes obtained from the STRING database using the GOSemSim R package. As shown in the histogram and cloud-rain diagram (Fig. 7A-B), the importance of the Sirt2 genes in the pathway was moderate. Since ST7 was not associated with related genes in the STRING database, it was not included in the friends analysis.

For the purpose of exploring the correlation between the 11 genes, we performed correlation analysis based on the expression profile data of these 11 genes in the GSE145226 dataset. As shown in Fig. 7C, except for Nampt and Nudt12, Nampt and Nnt, Nampt and Sirt5, Nnmt and Sirt5, Nnmt and Foxo1, Sirt2 and Pnp, Pnp and Ankle2, which are highly correlated, the rest of the genes have low correlations. In summary, the importance of both Sirt2 and Pnp in the pathway were moderate compared to other genes, and the biological functions of these two genes in the NP may be similar.

### Immune infiltration analysis of ARDEGs

**Fig. 8:**
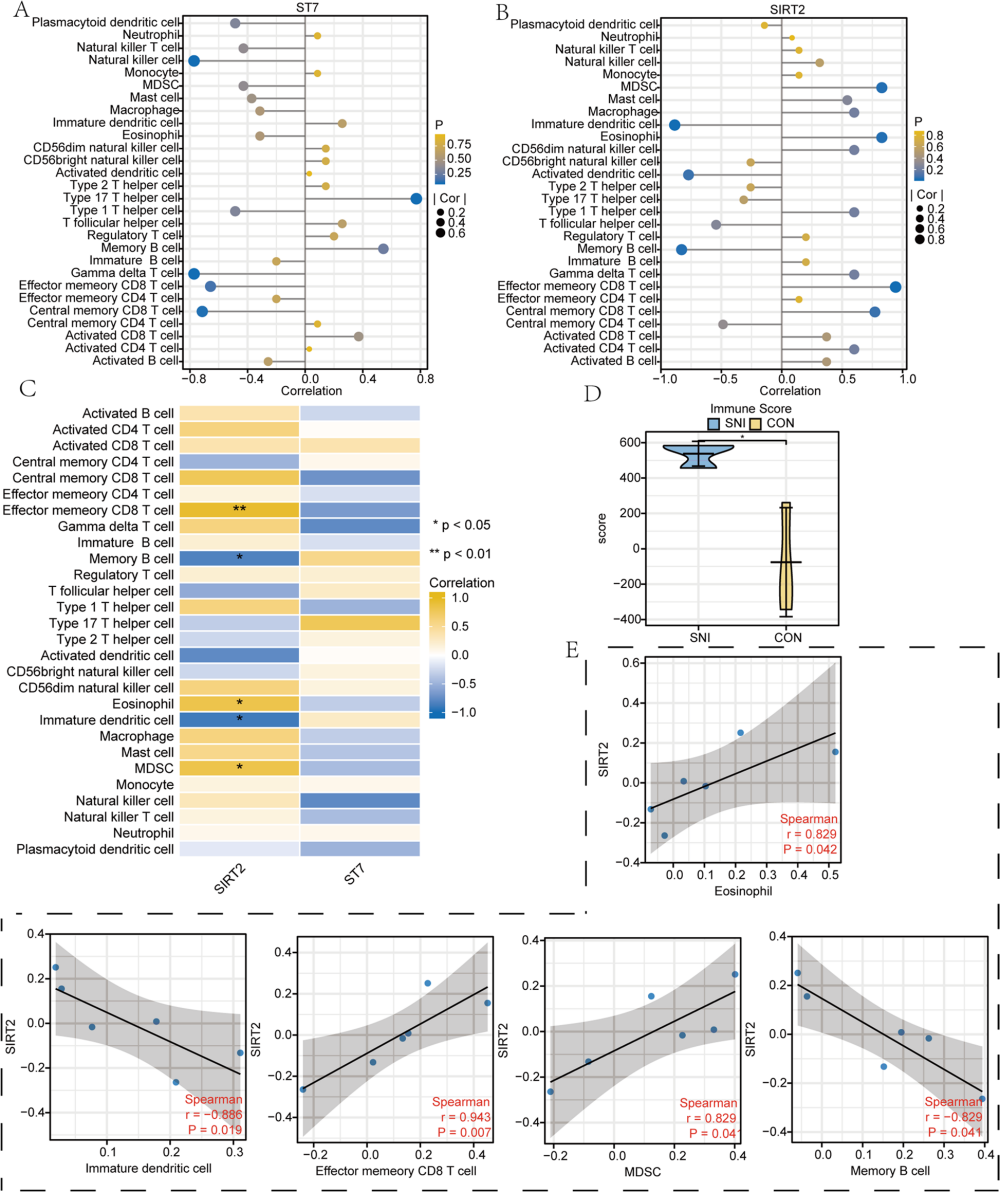
Immune infiltration analysis of ARDEGs. **A** Lollipop plot of the correlation analysis between the expression level of ST7 and the abundance of immune cell infiltration. **B** Lollipop plot of the correlation analysis between the expression level of Sirt2 and the abundance of immune cell infiltration.** C** The Correlation heatmap of correlation analysis between the expression level of ARDEGs and the abundance of immune cell infiltration. **D** The violin diagram of immune Score of different groups (SNI and control) in GSE145226. **E** Scatter plot of correlation analysis between Sirt2 and immune cell infiltration abundance

By using the ssGSEA algorithm to calculate the infiltration of 28 kinds of immune cells, we calculated the correlation between two ARDEGs (Sirt2, ST7) and the abundance of immune cell infiltration respectively. And lollipop plots were drawn to visualize the correlation results (Fig. 8A-B). Correlation heatmaps were then drawn based on the correlations and *p*-value between gene expression level and immune cell infiltration abundance (Fig. 8C).

Based on the expression profile data of the GSE145226 dataset, we used the estimate R package to obtain the immune score of genes, and drew a group comparison chart (Fig. 8D) [41]. The results showed that the immune score was statistically significant with different groups and illustrated that neuropathic pain was associated with high immune infiltration.

Finally, the correlation between the expression level of Sirt2 and the abundance of immune cell infiltration (Fig. 8E) was visualized. The results showed that the expression level of Sirt2 had a high positive correlation with the infiltration abundance of Eosinophil (*r *= 0.829, *P* = 0.042), Effector memory CD8^+^T cell (*r* = 0.943, *P* = 0.007), Myeloid-derived suppressor cell (MDSC) (*r* = 0.829, *P* = 0.041), and showed a high negative correlation with the infiltration abundance of Immature dendritic cell (*r* = -0.886, *P* = 0.019), and Memory B cell (*r* = -0.829, *P* = 0.041). In brief, the results of the immune infiltration analysis suggested that Sirt2 may be closely associated with neurological immune responses and involved in the regulation of immune-related signaling.

## Discussion

It is well known that autophagy is a biological process that specifically removes and recycles aggregated proteins and damaged organelles to maintain cell survival [42, 43]. Accumulating evidence suggests that nerve injury induced a significant upregulation of autophagy activation in damaged nerves, dorsal root ganglion neurons, the dorsal horn of the spinal cord, and plays an important role in the development of neuropathic pain [44, 45]. Elucidating the correlation between autophagy and neuropathic pain may provide new biomarkers and novel insights for the diagnosis and treatment of neuropathic pain.

In our study, we found 2 differentially expressed ARGs based on GSE145226 dataset and the GeneCards database, including Sirt2 and ST7. At present, researchers have shown that Sirt2 may play an important role in neuropathic pain, or served as a biomarker for neuropathic pain. A previous study had revealed that Sirt2 alleviates neuropathic pain by regulating NF-κB signaling and neuroinflammation [46]. Thus, Sirt2 may be a potential therapeutic target for the treatment of neuropathic pain. Xiaojiao Z et al. reported that Sirt2 inhibits ferroptosis in the spinal cord by upregulating FPN1 expression level, reducing lipid peroxidation caused by iron accumulation, and preserving changes in GPX4 and ACSL4 level, thereby alleviating NP [47]. However, there is currently no molecular mechanism by which Sirt2 is involved in the development of neuropathic pain by regulating autophagy. Another study revealed that Sirt2 may regulate cell proliferation and apoptosis by inhibiting autophagy [48]. And in the chronic constriction injury model, promotion of astrocytes autophagy and inhibition of apoptosis may be associated with remission of NP [49]. Therefore, we speculated that Sirt2 may regulate the progression of NP by suppressing autophagy. A previous study showed that ST7 was significantly negative correlation with level of the pro-inflammatory cytokine secretion [50]. Moreover, the activation of autophagy inhibits neuroinflammation, which in turn relieves neuropathic pain [13]. In sum, although there is no study on the involvement of ST7 in the occurrence and development of NP, we suppose that it is necessary to further investigate its potential functions in NP.

GO and KEGG enrichment analysis showed that two ARDEGs and their co-expressed genes were mainly related to pyridine nucleotide metabolic process, nicotinamide nucleotide metabolic process, Nicotinate and nicotinamide metabolism. In the GSEA results, we mainly found that NF-κB pathway, KRAS signaling, P53 pathway were associated with NP. Lin Z et al revealed that Astaxanthin alleviated neuropathic pain by inhibiting the nuclear factor-κB (NF-κB) p65 and the inflammatory response [51]. Overexpression of Fn14 activated the NF-κB pathway by promoting the translocation of p65 into the nucleus of damaged dorsal root ganglia (DRG) neurons, thereby promoting the progression of NP [52]. Gao Y et al also revealed that suppression of p53 resulted in down-regulation of p53 protein expression, thereby relieving thermal hyperalgesia in the chronic contractile injury model [53]. In a mouse model of spinal nerve ligation, GADD45A may play a role in the development of neuropathic pain by activating the p53 pathway [54]. Thus, these two ARDEGs may play vital roles in NP through these pathways.

Currently, researchers have revealed that TFs and microRNAs were crucial regulatory factors in the development of NP [46, 55]. Therefore, we constructed regulatory networks of TF-mRNA, microRNA-mRNA interactions, respectively, to reveal the potential interactions among the TFs, ARDEGs, and microRNA under pain conditions. As shown in Fig. 4, Cebpb was connected to two ARDEGs, suggesting its importance in NP. Zhisong Li reported that peripheral nerve injury caused by chronic contractile injury (CCI) upregulates the abundance of the transcription factor Cebpb in the dorsal root ganglion. Inhibition of this upregulation alleviated the development and maintenance of CCI-induced mechanical and thermal pain threshold hypersensitivity [56]. Therefore, Cebpb may become a new drug target of NP by regulating ARDEGs. So far, there is no direct evidence for the role of rno-miR-140-5p and rno-miR-877 in NP. However, miR-140-5p could target CLDN2 to promote cell viability and inhibit apoptosis, autophagy and inflammation in lipopolysaccharide induced sepsis model [57]. Thus, the miR-140-5p/Sirt2 axis may be involved in the occurrence and development of NP. In our study, we also found that several potential therapeutic compounds corresponding to Sirt2 and ST7 were identified for neuropathic pain. Among these potential therapeutic compounds, Valproic acid and Sirtinol deserved our attention. Valproic acid (VPA) is a recognized antiepileptic drug (AED). At present, AEDs are widely used in the treatment of NP, and they inhibit voltage-dependent sodium channels, increase serotonergic inhibition, reduce N-methyl-D-aspartate (NMDA) receptor-mediated glutamate excitation and enhance gamma aminobutyric acidergic (GABA) signaling for therapeutic effect [58]. Another study also revealed that VPA had good efficacy in the SNL model and equal potency in the inflammatory pain model [59]. Sirtinol is an inhibitor of the Sirtuin family of nicotinamide adenine dinucleotide (NAD)-dependent deacetylases in saccharomyces cerevisiae [60]. Ming et al revealed that administration of sirtinol following traumatic reduced cytokine production in male rats, which further could attenuate the inflammatory response [61]. Thus, sirtinol may be a potential compound for the relief of neuropathic pain, by inhibiting the inflammatory response, which may give us new insights into the treatment of neuropathic pain.

In the immune infiltration analysis results, we found that Sirt2 was positively correlated with Eosinophil, Effector memeory CD8^+^ T cell, myeloid-derived suppressor cells (MDSC). Furthermore, immune score was statistically significant with different groups, suggesting the changes of immune microenvironment in NP. Ifergan I et al. have revealed that effector memory CD8^+^ T lymphocytes were more inclined to migrate between blood–brain barrier endothelial cells than non-effector memory cells, which facilitated CD8^+^ T lymphocyte participation in immune responses in the central nervous system [62]. The exact role of CD8^+^ T lymphocytes in the central nervous system (CNS) inflammation remains controversial, but a study has revealed that CD8^+^ T cells are closely associated with demyelinated axons [63]. Another study has shown that Eosinophils are located near nerves during chronic inflammation and that activated eosinophils can induce nerve damage neuropeptide release [64]. P2X4, a sensitive purinergic receptor, is highly expressed on eosinophils. And P2X4 can regulate neuropathic pain through brain-derived neurotrophic factors and participate in the inflammatory in response to high ATP release [65]. Meanwhile, our study also found that Sirt2 was positively correlated with eosinophils, and the expression of Sirt2 was up-regulated in NP, suggesting that Sirt2 may be a promoter of neuroinflammation and may be involved in immune-related signal regulation.

There are some limitations that must be taken into account in the study. At first, autophagy-related genes were involved in our study may be incomplete. Furthermore, in vivo and in vitro experiments were not used to validate our results. Finally, non-tumor diseases often cannot perform clinical correlation studies and prognostic analyses due to lack of clinical and prognostic data. Despite these limitations, the present transcriptomic study can serve as an important molecular basis and provide reliable molecular biomarkers for the diagnosis and prognosis of NP. It also prepares for the exploration of new therapeutic targets for NP.

## Conclusions

In conclusion, we found that Sirt2 and ST7 may be potential therapeutic targets for treatment with neuropathic pain, providing more evidence about the crucial role of autophagy in neuropathic pain. And the miR-140-5p and Cebpb were important regulators at the post-transcriptional and transcriptional level in the mechanism of neuropathic pain. Immune cell infiltration may play a vital role in the development of neuropathic pain, especially Effector memory CD8^+^ T lymphocytes and Eosinophils. Our study may help to elucidate the pathogenesis of neuropathic pain. However, more research and experiments are needed to illustrate the functions of these molecules.

## Supplementary Information

**Additional file 1.**

**Additional file 2.**

**Additional file 3.**

## Data Availability

All data generated or analysed during this study are included in this published article and its supplementary information files.

## Abbreviations

NP: Neuropathic pain
ARDEGs: Autophagy-related differentially expressed genes
DE-miRNAs: Differentially expressed miRNAs
GO: Gene ontology
KEGG: Kyoto encyclopedia of genes and genomes
GSEA: Gene set enrichment analysis
TF: Transcription factor
ssGSEA: Single sample gene set enrichment analysis
SNI: Spared nerve injury
ARGs: Autophagy related genes
DEGs: Differentially expressed genes
PPI: Protein–protein interaction
BP: Biological process, MF: Molecular function
CC: Cell component
BH: Benjamini-Hochberg
MSigDB: Molecular signatures database
CTD: Comparative toxicogenomic database
CNS: Central nervous system
DRG: Dorsal root ganglia
GEO: Gene expression omnibus
MDSC: Myeloid-derived suppressor cells

## References

[1] Wu S, Bono J, Tao Y (2019). Long Noncoding RNA (lncRNA): A target in neuropathic pain. Expert Opin Ther Tar.

[2] Ellis A, Bennett DLH (2013). Neuroinflammation and the generation of neuropathic pain. Brit J Anaesth.

[3] Colloca L, Ludman T, Bouhassira D, Baron R, Dickenson AH, Yarnitsky D, et al. Neuropathic pain. Nat Rev Dis Primers. 2017;3:17002. 10.1038/nrdp.2017.2 PMC5371025 28205574

[4] Sayo A, Konishi H, Kobayashi M, Kano K, Kobayashi H, Hibi H, et al. GPR34 in spinal microglia exacerbates neuropathic pain in mice. J Neuroinflamm. 2019;16:82. 10.1186/s12974-019-1458-8 PMC6458787 30975169

[5] Levine B, Kroemer G (2008). Autophagy in the pathogenesis of disease. Cell.

[6] Deretic V (2011). Autophagy in immunity and cell-autonomous defense against intracellular microbes. Immunol Rev.

[7] Goldshmit Y, Kanner S, Zacs M, Frisca F, Pinto AR, Currie PD (2015). Rapamycin increases neuronal survival, reduces inflammation and astrocyte proliferation after spinal cord injury. Mol Cell Neurosci.

[8] Pan R, Timmins GS, Liu W, Liu KJ (2015). Autophagy mediates astrocyte death during zinc-potentiated ischemia–reperfusion injury. Biol Trace Elem Res.

[9] Kocak M, Ezazi Erdi S, Jorba G, Maestro I, Farrés J, Kirkin V (2022). Targeting autophagy in disease: established and new strategies. Autophagy.

[10] Shi CS, Shenderov K, Huang NN, Kabat J, Abu-Asab M, Fitzgerald KA (2012). Activation of autophagy by inflammatory signals limits IL-1β production by targeting ubiquitinated inflammasomes for destruction. Nat Immunol.

[11] Moalem G, Tracey DJ (2006). Immune and inflammatory mechanisms in neuropathic pain. Brain Res Rev.

[12] Thacker MA, Clark AK, Marchand F, McMahon SB (2007). Pathophysiology of peripheral neuropathic pain: immune cells and molecules. Anesth Analg.

[13] Shi G, Shi J, Liu K, Liu N, Wang Y, Fu Z (2013). Increased miR-195 aggravates neuropathic pain by inhibiting autophagy following peripheral nerve injury. Glia.

[14] Wang O, Chin R, Cheng X, Wu MKY, Mao Q, Tang J (2019). Efficient and unique cobarcoding of second-generation sequencing reads from long DNA molecules enabling cost-effective and accurate sequencing, haplotyping, and de novo assembly. Genome Res.

[15] Tang S, Jing H, Huang Z, Huang T, Lin S, Liao M (2020). Identification of key candidate genes in neuropathic pain by integrated bioinformatic analysis. J Cell Biochem.

[16] Guida F, Iannotta M, Misso G, Ricciardi F, Boccella S, Tirino V (2022). Long-term neuropathic pain behaviors correlate with synaptic plasticity and limbic circuit alteration: A Comparative Observational Study in Mice. Pain.

[17] Barrett T, Wilhite SE, Ledoux P, Evangelista C, Kim IF, Tomashevsky M (2012). NCBI GEO: Archive for Functional Genomics Data Sets—Update. Nucleic Acids Res.

[18] Davis S, Meltzer PS (2007). GEOquery: A Bridge Between the Gene Expression Omnibus (GEO) and BioConductor. Bioinformatics.

[19] Shi Y, Zhang X, Fang Q, Zhan H, Wang X, Huang X, et al. LANCL1 as the key immune marker in neuropathic pain. Neural Plast. 2022;2022:9762244. 10.1155/2022/9762244 PMC9061068 35510269

[20] Zhou X, Du J, Liu C, Chen Y, Liu L, Wu D (2021). A pan-cancer analysis of CD161, a Potential New Immune Checkpoint. Front Immunol..

[21] Ritchie ME, Phipson B, Wu D, Hu Y, Law CW, Shi W (2015). Limma powers differential expression analyses for RNA-sequencing and microarray studies. Nucleic Acids Res.

[22] Deng Y, He W, Cai H, Jiang JH, Yang YY, Dan YR (2022). Analysis and validation of Hub genes in blood monocytes of postmenopausal osteoporosis patients. Front Endocrinol.

[23] Zhou J, Xiong W, Wang Y, Guan J (2021). Protein function prediction based on PPI networks: Network Reconstruction Vs Edge Enrichment. FrontGenet.

[24] Szklarczyk D, Gable AL, Nastou KC, Lyon D, Kirsch R, Pyysalo S (2021). The STRING database in 2021: Customizable Protein-Protein Networks, and Functional Characterization of User-Uploaded Gene/Measurement Sets. Nucleic Acids Res.

[25] Ramadhani HF, Annisa A, Tedjo A, Noor DR, Kusuma WA (2022). Combination of Enrichment Using Gene Ontology and Transcriptomic Analysis Revealed Contribution of Interferon Signaling to Severity of COVID-19. Interdiscip Perspect Infect Dis.

[26] Pan Q, Zhou R, Su M, Li R (2019). The Effects of Plumbagin on Pancreatic Cancer: A Mechanistic Network Pharmacology Approach. Med Sci Monit.

[27] Shannon P, Markiel A, Ozier O, Baliga NS, Wang JT, Ramage D (2003). Cytoscape: A Software Environment for Integrated Models of Biomolecular Interaction Networks. Genome Res.

[28] Gene Ontology Consortium (2015). Going Forward. Nucleic Acids Res.

[29] Kanehisa M (2000). KEGG: Kyoto Encyclopedia of Genes and Genomes. Nucleic Acids Res.

[30] Yu G, Wang L, Han Y, He Q (2012). Clusterprofiler: An R Package for Comparing Biological Themes Among Gene Clusters. OMICS.

[31] Subramanian A, Tamayo P, Mootha VK, Mukherjee S, Ebert BL, Gillette MA (2005). Gene set enrichment analysis: A Knowledge-Based Approach for Interpreting Genome-Wide Expression Profiles. Proc Natl Acad Sci.

[32] Liberzon A, Birger C, Thorvaldsdóttir H, Ghandi M, Mesirov JP, Tamayo P (2015). The Molecular signatures database hallmark gene set collection. Cell Syst.

[33] Zhou K, Liu S, Sun W, Zheng LL, Zhou H, Yang JH (2017). ChIPBase V2.0: Decoding Transcriptional Regulatory Networks of Non-Coding RNAs and Protein-Coding Genes From ChIP-seq Data. Nucleic Acids Res.

[34] Zhang Q, Liu W, Zhang HM, Xie GY, Miao YR, Xia M (2020). HTFtarget: A Comprehensive Database for Regulations of Human Transcription Factors and their Targets. Genomics Proteomics Bioinformatics.

[35] Ru Y, Kechris KJ, Tabakoff B, Hoffman P, Radcliffe RA, Bowler R (2014). The multiMiR R Package and Database: Integration of microRNA–Target Interactions Along with their Disease and Drug Associations. Nucleic Acids Res.

[36] Peng P, Zhang B, Huang J, Xing C, Liu W, Sun C (2020). Identification of a circRNA-miRNA-mRNA network to explore the effects of circRNAs on pathogenesis and treatment of spinal cord injury. Life Sci.

[37] Davis AP, Grondin CJ, Johnson RJ, Sciaky D, Wiegers J, Wiegers TC (2021). Comparative Toxicogenomics Database (CTD): Update 2021. Nucleic Acids Res.

[38] Yu G (2020). Gene Ontology semantic similarity analysis using GOSemSim.

[39] Xiao B, Liu L, Li A, Xiang C, Wang P, Li H (2020). Identification and verification of immune-related gene prognostic signature based on ssGSEA for osteosarcoma. Front Oncol.

[40] Guo A, Li J, Luo L, Chen C, Lu Q, Ke J, Feng X (2021). Valproic acid mitigates spinal nerve ligation-induced neuropathic pain in rats by modulating microglial function and inhibiting neuroinflammatory response. Int Immunopharmacol.

[41] Tennant PWG, Arnold KF, Ellison GTH, Gilthorpe MS. Analyses of 'change scores' do not estimate causal effects in observational data. Int J Epidemiol. 2022;51:1604–15. 10.1093/ije/dyab050 PMC9557845 34100077

[42] Glick D, Barth S, Macleod KF (2010). Autophagy: cellular and molecular mechanisms. J Pathol.

[43] Chen H, Zhou C, Xie K, Meng X, Wang Y, Yu Y (2019). Hydrogen-rich saline alleviated the hyperpathia and microglia activation via autophagy mediated inflammasome inactivation in neuropathic pain rats. Neuroscience.

[44] Liu X, Zhu M, Ju Y, Li A, Sun X (2019). Autophagy dysfunction in neuropathic Pain. Neuropeptides.

[45] Cai W, Zhang Y, Su Z (2020). CiRS-7 targeting miR-135a-5p promotes neuropathic pain in CCI rats via inflammation and autophagy. Gene.

[46] Zhang Y, Chi D (2018). Overexpression of SIRT2 alleviates neuropathic pain and neuroinflammation through deacetylation of transcription factor nuclear factor-Kappa B. Inflammation.

[47] Zhang X, Song T, Zhao M, Tao X, Zhang B, Sun C (2022). Sirtuin 2 alleviates chronic neuropathic pain by suppressing ferroptosis in rats. Front Pharmacol.

[48] Liu S, Gao X, Fan Z, Wang Q. SIRT2 Affects cell proliferation and apoptosis by suppressing the level of autophagy in renal podocytes. Dis Markers. 2022;2022:4586198. 10.1155/2022/4586198 PMC9054447 35493297

[49] Jin G, Yue R, He S, Hong L, Xu Y, Yu C (2018). Koumine decreases astrocyte-mediated neuroinflammation and enhances autophagy, contributing to neuropathic pain from chronic constriction injury in rats. Front Pharmacol.

[50] Liu H, Lu W, He H, Wu J, Zhang C, Gong H (2019). Inflammation-dependent overexpression of c-Myc enhances CRL4DCAF4E3 ligase activity and promotes ubiquitination of ST7 in colitis-associated cancer. J Pathol.

[51] Zhao L, Tao X, Song T (2021). Astaxanthin alleviates neuropathic pain by inhibiting the MAPKs and NF-κB pathways. Eur J Pharmacol.

[52] Huang L, Zou Y, Wu S, Zhang HH, Mao QX, Li JB (2019). Fn14 Participates in neuropathic pain through NF-κB pathway in primary sensory neurons. Mol Neurobiol.

[53] Gao Y, Sun N, Wang L, Wu Y, Ma L, Hong J (2018). Bioinformatics analysis identifies p53 as a candidate prognostic biomarker for neuropathic pain. Front Genet.

[54] Li J, Chen J (2022). GADD45A induces neuropathic pain by activating P53 apoptosis pathway in mice. Genes Genom.

[55] Jiangpan P, Qingsheng M, Zhiwen Y, Tao Z (2016). Emerging role of microRNA in neuropathic pain. Curr Drug Metab.

[56] Li Z, Mao Y, Liang L, Wu S, Yuan J, Mo K, et al. The transcription factor C/EBPβ in the dorsal root ganglion contributes to peripheral nerve trauma–induced nociceptive hypersensitivity. Sci Signal. 2017;10:eaam5345. 10.1126/scisignal.aam5345 PMC5665022 28698219

[57] Han D, Fang R, Shi R, Jin Y, Wang Q (2021). LncRNA NKILA knockdown promotes cell viability and represses cell apoptosis, autophagy and inflammation in lipopolysaccharide-induced sepsis model by regulating miR-140–5p/CLDN2 Axis. Biochem Bioph Res Co.

[58] Winkler I, Blotnik S, Shimshoni J, Yagen B, Devor M, Bialer M (2005). Efficacy of antiepileptic isomers of Valproic Acid and Valpromide in a rat model of neuropathic pain. Brit J Pharmacol.

[59] Kaufmann D, West PJ, Smith MD, Yagen B, Bialer M, Devor M (2017). Sec -Butylpropylacetamide (SPD), a new amide derivative of Valproic Acid for the treatment of neuropathic and inflammatory pain. Pharmacol Res.

[60] Cai AL, Zipfel GJ, Sheline CT (2006). Zinc neurotoxicity is dependent on intracellular NAD levels and the sirtuin pathway. Eur J Neurosci.

[61] Liu FC, Day YJ, Liou JT, Lau YT, Yu HP (2008). Sirtinol attenuates hepatic injury and pro-inflammatory cytokine production following trauma-hemorrhage in male Sprague-Dawley rats. Acta Anaesthesiol Scand.

[62] Ifergan I, Kebir H, Alvarez JI, Marceau G, Bernard M, Bourbonnière L (2011). Central nervous system recruitment of effector memory CD8+ T lymphocytes during neuroinflammation is dependent on α4 integrin. Brain.

[63] Neumann H (2002). Cytotoxic T lymphocytes in autoimmune and degenerative CNS diseases. Trends Neurosci.

[64] Drake MG, Scott GD, Blum ED, Lebold KM, Nie Z, Lee JJ, et al. Eosinophils increase airway sensory nerve density in mice and in human asthma. Sci Transl Med. 2018;10:eaar8477. 10.1126/scitranslmed.aar8477 PMC6592848 30185653

[65] Paalme V, Rump A, Mädo K, Teras M, Truumees B, Aitai H (2019). Human peripheral blood eosinophils express high levels of the purinergic receptor P2X4. Front Immunol.

